# Dr. Kinglake's Remarks on Angustura Bark

**Published:** 1807-05

**Authors:** Robert Kinglake

**Affiliations:** Taunton


### *To the Editors of the Medical and Physical Journal.*

Gentlemen,

In the last Number of the Medical and Chirurgical Review is an article of intelligence, relative to a poisonous species of Angustura bark having been vended at Hamburgh. If what has recently occurred in this town from the use of a drug sold under the appellation of Angustura bark, should also have happened in other places, the evil of having such a pernicious substance on sale is of a most threatening nature, and ought, without delay, to be exposed and obviated.

In five instances within these three years, a drug sold in this town for Angustura bark, and administered as such to four different persons, evidently produced the most distressing effects. In four of the instances alluded to, an approach to syncope, accompanied with more or less of universal tremor and spasmodic twitchings, are said to have occurred. In the fifth instance referred to, it was exhibited in a case of low remittent fever, and was speedily followed by effects similar to those which presented in the other instances. On this occasion, however-death, death ensued; but whether that event be justly attributable to the influence of the medicine or that of the disease, is doubtful. In each of the other instances the patients suffered severely from the ailment induced, during some hours, and its hurtful effects endured several days.

In the article of intelligence recorded in the Medical and Chirurgical Review, respecting the true and spurious sorts of Angustura bark, it is stated, that "they may be distinguished by the following characters: the decoction of the true Angustura bark dyes linen yellow, is not turbid, nor is altered by a solution of iron: that of the spurious kind does not dye linen, and becomes black with chalybeates." With a view to ascertain whether the quality of the Angustura bark, sold in the shops of this town, could be detected by the respective tests here cited, I procured a specimen of the drug from three different druggists residing in this place. A separate decoction was made of the several parcels, and I regret to add, that each of them clearly denoted what is affirmed to be the distinguishing characters of the spurious and poisonous sort of the drug. To pieces of linen infused in each decoction, was imparted a dusky hue, in which the yellow colour, said to be characteristic of the true species was scarcely perceptible; each decoction was also turbid and deeply blackened by the admixture of a solution of iron.

If these are unequivocal tests of the noxious kind of Angustura bark, it surely becomes incumbent on the College of Physicians, or on some other adequate authority, immediately to institute such an investigation of the subject, as may hereafter exempt mankind from the deleterious effects of a *spurious* drug, which, in the full confidence of its being *genuine*, is very commonly directed as a mild and salutary tonic.

The inquiry indeed appears to be additionally necessary, in as far as Angustura bark is usually resorted to in cases of chronic weakness from various causes, and more particularly in the convalescent periods of febrile affection. When exhibited under these circumstances, it is difficult correctly to mark its effects. The symptoms of the disease it is then directed to remedy, so much resemble those of its own hurtful operation, that any aggravation of complaint occurring during its employ, would be naturally imputed rather to an unfavourable course of the disease than to any conceivable injury from the medicine. Under this ambiguity of effect, therefore, it is not extraordinary that

 the the injurious agency or a spurious sort of Angustura bark should have escaped general notice.

Further research into the subject will probably furnish additional criteria, by which the true sort of Angustura bark may be distinguished from that which is spurious, with more promptness and certainty than can at present be done. It must be allowed to be an object of no small concern to the public welfare, to have a drug, like Angustura bark, high in both medical and popular estimation, liable to be *counterfeited* by an article, said to "operate as a poison," and which, in the several instances here adduced, did actually produce effects truly alarming.

I am, &c.

*Taunton, Feb.* 13, 1807. ROBERT KINGLAKE.

